# Supplementary material for: Wearable neurofeedback acceptance model for students’ stress and anxiety management in academic settings
Source: PLoS One. 2024 Oct 24;19(10):e0304932. doi: 10.1371/journal.pone.0304932 (PMC11501020; doi:10.1371/journal.pone.0304932)
Supplement: Attachment — Submitted filename: Response to Reviewers.docx [file pone.0304932.s001.docx]

Response to Editorial Office Comment of the entitled manuscript:

**"Wearable neurofeedback acceptance model for students' stress and anxiety management in academic settings"**

Dear Editor in Chief Dr. Chenette,

Please find below our response regarding the Editorial Office comment.

**Comment**

Thank you very much for submitting your manuscript to PLOS ONE, and for responding to our recent requests regarding your submission. After careful evaluation, we have decided that your submission does not meet our publication criteria and must be rejected. Based on our evaluation of your manuscript and the information you provided, we do not feel that your submission meets our ethical requirements for human subjects research submissions. PLOS ONE requires that research meets all applicable standards for the ethics of experimentation and research integrity (http://journals.plos.org/plosone/s/human-subjects-research). We reserve the right to reject any submission that does not meet our internal ethical standards, which in some cases are more stringent than local ethical standards. We recognize that you obtained ethical approval from your local ethics committee after you began your study: your ethics approval document is dated 18 March 2024, while you began collecting patient samples from Aug to Nov 2022. You also state that informed consent was obtained from all participants, which indicates that the samples were prospectively collected for research purposes. For human subjects research, PLOS ONE requires that authors obtain ethics committee approval before beginning the study, and we do not accept retrospective ethical approval. As a result of these concerns we cannot consider the manuscript for publication at PLOS ONE. I am sorry that we cannot be more positive on this occasion, but hope that you understand the reasons for this decision.

**Response:** We fully understand the policy of PLOS ONE journal regarding the Ethics, and in this vein we are attaching the updated IRB (original) and updated manuscript file that comply with the ethical standards. The misunderstanding from our side was the reported year in the manuscript and the attachment of the extension of the study instead of the original IRB approval. In this vein, we have corrected the following information in the manuscript (line 260):

*“Between August and November 2023,…”*
