# Supplementary material for: Wearable neurofeedback acceptance model for students’ stress and anxiety management in academic settings
Source: PLoS One. 2024 Oct 24;19(10):e0304932. doi: 10.1371/journal.pone.0304932 (PMC11501020; doi:10.1371/journal.pone.0304932)
Supplement: Attachment — Submitted filename: PONE-D-24-19965_Response to Reviewers.docx [file pone.0304932.s002.docx]

Response to Editorial Office Comment of the entitled manuscript [PONE-D-24-19965]:

**"Wearable neurofeedback acceptance model for students' stress and anxiety management in academic settings"**

Dear Editor in Chief Dr. Chenette and Academic Editor Dr. Manob Jyoti Saikia,

We would like to thank you and the reviewers for the constructive comments. We have followed the suggestions and revised the manuscript accordingly. A marked-up copy of our manuscript that highlights changes made to the original version is uploaded in companion to this rebuttal letter and a cleaned version of the revised manuscript. A detailed description of our response to the revision comments follows.

**Reviewer 1**

Manuscript is well written. However some informations are found to be missing.

**Comment 1**: The work incorporates EEG & other biosignals to develop the neuro feedback system. The sampling rate of acquired signals, location of placing electrodes, frequency band of EEG used for the neuro feedback system are unknown.

- *Response*: We thank the reviewer for their comment. To address it, we have added the following text (pg. 6) that explains the missing sampling frequency and the location of the electrodes:

*The EEG electrodes are placed according to the 10/20 international system, employing monopolar at Cz site and bipolar recording, where, in the latter case, the potential difference is measured between electrodes at the sites of Fp1/Fp2 snd T3/T4 (or C3/C4). The reference electrode is placed behind the right earlobe. The sampling frequency allows for EEG spectrum up to 250Hz which covers all the related EEG bands. The fNIRS electrodes are place bilaterally at the forehead (3 cm from the Fp1 and Fp2 sites, respectively). The BVP sensor is placed with a clip in the ear, and the TEMP sensor is mounted on the forehead between the Fp1 and Fp2 locations. The EDA sensor is placed on the index (+) and middle finger (-) of the right hand.*

With regards to the frequency bands comment, we mention in the added text that the sampling frequency allows for EEG spectrum up to 250Hz covering all EEG bands. In the next page (pg. 7) there was from the original submission an analytical description of the EEG bands related with the stress as neurofeedback target. For convenience, we copy the text again here:

*EEG, a common signal for brain activity, is widely used in stress detection and evaluation [53]. Changes in EEG frequency bands (Delta (0.5 – 4 Hz), Theta (4 – 8 Hz), Alpha (8 – 13 Hz), Beta (14 – 30 Hz), and Gamma (30-80Hz)) correlate with stress levels. Altered rhythms, like increased delta in challenges, elevated theta in stress, suppressed alpha, and varied beta in task difficulty, offer insights into mental stress and psychological disorders [54].*

**Comment 2**: Preprocessing of biosignals is not given much importance in the manuscript.

- *Response*: According to the comment, the following text was added in pg. 8 in the section of **Biosignals pre-processing and analysis modules**

*In particular, down-sampling reduces the sampling rate of the signal, which can help in reducing the amount of data and computational load, being particularly useful when the original sampling rate is higher than necessary for the analysis. The bandpass filters isolate the relevant components of the biosignal and remove the noise. For example, in ECG analysis, bandpass filters can remove low-frequency baseline wander and high-frequency muscle noise [59]. Moreover, moving averages technique smooths the signal by averaging a set number of data points. It helps in reducing random noise and making the underlying trends in the signal more apparent by removing baseline modulation and artifacts. Artifact removal techniques like Independent Component Analysis (ICA) [60] and adaptive filtering [61] are used to identify and remove artifacts from biosignals. ICA, for instance, can separate mixed signals into independent components, allowing for the removal of artifacts like eye blinks in EEG signals [62]. Finally, after removing noise and artifacts, normalization is performed to scale the signal to a standard range, making it easier to compare and analyze.*

The references [60, 61, 62] were added, accordingly:

[60] Hyvärinen A. Independent component analysis: recent advances. Philos Trans A Math Phys Eng Sci. 2012;371(1984):20110534.

[61] Thannoon HH, Hashim IA. Efficient enhanced Recursive Least Square algorithm adaptive filtering scheme for artifacts removal in ECG signals. e-Prime-Advances in Electrical Engineering, Electronics and Energy. 2023;6:100318.

[62] Rashida M, Habib MA. Feature Extraction from Regenerated EEG–A better approach for ICA based eye blink artifact detection. Frontiers in Biomedical Technologies. 2024;11(2):199-206.

**Comment 3**: Temperature is also considered for the feedback system. But there is no information how body temperature relates stress or anxiety.

- *Response*: As we mention in our response to the first comment, in pg. 7 of the original manuscript there is a description about the way the temperature is connected with the stress or anxiety. For convenience, we copy the text again here:

*Under acute stress, sympathetically-mediated vasoconstriction causes a rapid drop in skin temperature; hence TEMP signals dynamic changes that can be used to reflect acute stress effects [58].*

**Comment 4**: Secondly, the work uses people from different age groups. Number of participant per group is not available. But output responses are average out. Age might be an important factor when stress/anxiety is quantised.

- *Response*: In the original manuscript, Table 1 shows the number of participants per group. Moreover, Table 10 summarizes the effects of Multi grouping analysis, including the path coefficient effects from Age Groups. As it is stated in pg. 18:

*A specific effect on PEoU🡪UA and PU🡪UA is noticed for the age differences {AGE1: 18-24yrs, AGE3: 35-44yrs} and {AGE2: 25-34yrs, AGE3: 35-44yrs}.*

Nevertheless, as it is concluded in pg. 19:

*Clearly, these could be considered as quite specific effects that do not affect the generality of the mSense TAM framework, as shown from the results in Tables 2-10.*

**Comment 5**: Thirdly, how the level of stress correlates with the TEM model?

- *Response*: We believe that the term “TEM model” has been misspelled from the “TAM model”. Based on this, in the qualitative analysis findings (pg. 19) the users expressed significant perceived usefulness, usage attitude leading to intention to use for managing stress and anxiety. These views, actually, express the constructs of the TAM model. As we extensively discuss in the implications, limitations and future work section (pg. 25/27) *“the propose mSense TAM serves as a foundational structure that can accommodate additional constructs, allowing a detailed understanding of wearable framework adoption from end-users' perspectives across different technologies, user types, and usage settings.”* (pg. 25). From this perspective, in case we want to examine the specific role of stress level within the TAM model, the stress level could be considered as an external variable and be included in the multigroup analysis (as in Table 10). Since we did not have clinical validation for the stress/anxiety level of each participant, we did not include any related variable for the validity and consistency of our approach. Nevertheless, we consider the reviewer’s comment as a helpful suggestion for future studies that would target clinically diagnosed patients with stress or anxiety.

**Comment 6**: Overall representation is found to be good.

- *Response*: We thank the reviewer for their positive overall evaluation.
