# Supplementary material for: Wearable neurofeedback acceptance model for students’ stress and anxiety management in academic settings
Source: PLoS One. 2024 Oct 24;19(10):e0304932. doi: 10.1371/journal.pone.0304932 (PMC11501020; doi:10.1371/journal.pone.0304932)
Supplement: Attachment — Submitted filename: PONE-D-24-19965R2_Response to Reviewers.docx [file pone.0304932.s003.docx]

Response to Editorial Office Comment of the entitled manuscript [PONE-D-24-19965R2]:

**"Wearable neurofeedback acceptance model for students' stress and anxiety management in academic settings"**

Dear Editor in Chief Dr. Chenette and Academic Editor Dr. Manob Jyoti Saikia,

We would like to thank you and the reviewers for the constructive comments. We have followed the suggestions and revised the manuscript accordingly. A marked-up copy of our manuscript highlighting changes made to the original version is uploaded in companion to this rebuttal letter, along with a cleaned version of the revised manuscript. A detailed description of our response to the revision comments follows.

**Reviewer #1**

**Comment 1:** Review comments are well addressed. The manuscript covers all the details of the experiment and the results are well explained.

- *Response*: We thank the reviewer for their comment.

**Reviewer #2**

**Comment 1:** The manuscript investigates the acceptance of a conceptual multimodal wearable sensing framework, mSense, which aims to give real-time neurofeedback for stress and anxiety management in students. As such, they distributed an online survey to individuals above 18 years of age to assess the perceived usefulness (PU), perceived ease of use (PEoU), usage attitude (UA), and intention to use (IU) on this conceptual technology. While most of the manuscript is well written, I feel there is a major problem with the Study Design section. There is very little information about the participants, their background in multimodal biosensor or wearable tech, and how much - if any - briefing they received about the proposed conceptual technology prior to completing the survey.

- *Response*: We thank the reviewer for their constructive feedback. We acknowledge the concern regarding the information provided about participants and their familiarity with multimodal biosensor or wearable technology. We appreciate the concern raised regarding the Study Design section, the information provided about the participants, and their familiarity with multimodal biosensor or wearable technology. From this perspective, we would like to clarify the following aspects:

We collected relevant demographic information, including gender, age, education level, and occupation. In particular, participants' occupations included Researchers/Professors, Software Developers/Engineers, Medical Doctors/Healthcare Professionals, and Biomedical Engineering Students. All of them are acquainted with commercial healthcare wearables, such as smartwatches, as they are increasingly utilized to monitor patient health, rapidly diagnose disease, and help predict and often improve patient outcomes. Moreover, all occupations in the survey relate to the areas of healthcare practice and research, biomedical engineering, and software development for healthcare; hence, the participants were aware of the type of biosignals usually collected by a healthcare wearable technology, e.g., EEG, ECG, BPM, EDA, etc, either separately or simultaneously. In most cases, incremental learning is activated when trying to explore a device (e.g., wearables), i.e., there is no need to know everything at once; the basic functions are prioritized, and, gradually, more advanced features can be explored when becoming more comfortable. In this vein, participants were informed before completing the survey, ensuring they had adequate context on mSense, by including the following information:

- Overview of the mSense Project: “The mSense project, funded by Khalifa University, aims to develop a non-obtrusive multimodal biosignal device and evaluate a deep learning AI model that integrates various biosignals, such as EEG, pulse rate, and electrodermal activity.”
- Description of the mSense Multisensor Headset: A thorough introduction with visuals of the mSense, describing the capabilities of the mSense multisensor headset system, including its ability to record EEG, ECG, EMG, respiration, skin temperature, and electrodermal activity (as shown in Figure 1 of the online survey).

Finally, from the results perspective, the subgrouping analysis results in Table 10 show no significant influence of diversity in occupation (background) on the paths of the mSense constructs.

**Comment 2:** P 5, L127: Please provide a citation for mTAM.

- *Response*: We thank the reviewer for their comment. The citation for mTAM is indeed provided in the text (specifically, reference [49] on page 5). To clarify this for the reader, we have moved reference [49] to follow the mention of mTAM, as shown below immediately:

*More recent modifications, such as mTAM [49], incorporate perceived novelty and perceived personalization constructs to explain user perspectives when adopting AI-based applications for behavior change.*

**Comment 3:** P6: I understand that this is a conceptual framework, but as a researcher who does multimodal biosensor work, I wonder how feasible it is to set up, connect, and synchronize all of these biosensors without professional help. This hugely affects usability and PEoU and potentially IU. Please include technical feasibility of the application/use of this device by novice users. Please also indicate whether the participants were briefed on the setup of the device as it is crucial in understanding whether they were able to effectively evaluate PEoU, UA, and IU.

- *Response*: We thank the reviewer for their comment. The proposed mSense hardware was based on the collaboration of Khalia University, the Department of Biomedical Engineering, and the private company PLUX Biosignals (<https://www.pluxbiosignals.com>), which specializes in biosignals sensing devices. The PLUX Biosignals company has huge experience producing biosensors and wearables (as you can see from their list and description of products and the award-winning Bitalino central data processor). Due to its extensive experience, we have selected PLUX Biosignals to ensure the highest technical integrity in the design, implementation, and UX of mSense. There were many co-creation meetings between Khalifa University and PLUX Biosignals to clearly define the needful biosignals and how even novice users could easily set them up. The final version of the mSense presented within the study works like wearing a cap. The sensing electrodes and the Bitalino processor have fixed positions that the user does not alter, as they are embedded within the cap. By turning mSense on, an automation process checks for the correct cap fitting and establishes Bluetooth connection, synchronization, correct data acquisition, and wireless storage. The mSense cap-shaped all-in-one device is visualized in the introduction (Figure 1 of the survey) to showcase to the participant the distribution of the electrodes and the way someone wears it.

**Comment 4:** P 6, L163: clip in the ear should be clip on the ear, I presume?

- *Response*: We thank the reviewer for their comment. The suggested change has been made, as follows:

*The BVP sensor is placed with a clip on the ear, and the TEMP sensor is mounted on the forehead between the Fp1 and Fp2 locations.*

**Comment 5:** Given that their analysis is based on participants evaluating a conceptual framework, I would have liked more details about the types of feedback given to the individuals, how it is delivered, when is it delivered (do they only receive feedback when they feel stress/anxiety?), if there are any delays (as there usually are in most types of real-time neurofeedback). Did the participants receive any details about the feedback given? (I did not see any information on the survey).

- *Response:* We thank the reviewer for their comment. The mSense framework evaluated in this study is designed to offer real-time feedback on stress and anxiety levels. During the online survey, participants were asked to provide their feedback on the mSense device's conceptual framework, including their perceptions and expectations regarding feedback. This was reflected in the Q6:

“Imagine you are using the mSense multisensory headset system, how useful would the following features be (from Extremely useful to Not at all usefulness):

1. Real time feedback regarding the stress level.

2. Real time feedback regarding the anxiety levels.

3. Feedback of the respiration and skin temperature.

The responses were incorporated within the PU construct of the mTAM model. Moreover, as the reviewer mentions, this is a conceptual evaluation; hence, the online survey did not specifically address these potential delays, but the feedback system's design aims to be as responsive as possible. The online survey aimed to collect high-level feedback on user preferences and potential improvements rather than detailed operational aspects.

**Comment 6:** Page 11, L 280: They state that they invited 300 mental health experts, doctors/health professionals, researchers, biomedical students, and designers/developers to participate in the survey research. Were all participants familiar with all of the biosensors? I looked at the survey and there is no description of any of the biosensors used. How do we know that they understand what these biosensors do and how to set them up successfully and use them? If they do not know how to use these biosensors, how could they rate their PEoU, UA, or IU?

- *Response*: Please see our response to Comment 1.

**Comment 7:** Page 12: Please provide upper range of participants’ age.

- *Response*: This information was omitted from the original submission since it was not considered crucial for the multigroup analysis, as it is mentioned in the caption of Table 10:

“AGE1: 18-24yrs; AGE2: 25-34yrs; AGE3: 35-44yrs (rest age ranges did not include significant number >9 to be included)…”

Nevertheless, to accommodate the request from the reviewer, the upper range of participants’ age has been added to Table 1 (page 12), as requested:

**Table 1. Demographic profile of the participants (**$\boldsymbol{n=106}$**).**

| **Item** |  | **Frequency** | **Percentage (%)** |
| --- | --- | --- | --- |
| Gender | Male | 43 | 40.6 |
|  | Female | 63 | 59.4 |
| Age | 18-24 | 44 | 41.5 |
|  | 25-34 | 33 | 31.1 |
|  | 35-44 | 15 | 14.2 |
|  | 45-54 | 9 | 8.5 |
|  | 55-64 | 3 | 2.8 |
|  | Above 65 | 2 | 1.9 |
| Education level | High School | 23 | 21.7 |
|  | Bachelors | 24 | 22.6 |
|  | Masters | 25 | 23.6 |
|  | PhD | 34 | 32.1 |
| Occupation | Researcher/Professor | 31 | 29.2 |
|  | Software/Game Developer | 13 | 12.3 |
|  | Healthcare Professional/Doctor | 10 | 9.4 |
|  | High Education Students | 52 | 49.1 |

**Comment 8:** Page 19~: It would be helpful if the authors broke down which of these qualitative responses were in response to which of the four open ended questions. For example, which ones were in response to the first open ended question: Please specify why would you not use the mSense system to monitor and manage your stress/anxiety levels.

- *Response*: We thank the reviewer for this insightful comment. In this line, we have coded the related open-ended questions as QEQ1-QEQ1 in the Qualitative Analysis section (page 14). Hence, using such coding, we have included the connection between the response and the related open-ended question in all responses. Additionally, the most relevant examples concerning OEQ1 (why participants might not use the mSense system to monitor and manage stress/anxiety levels) have been incorporated into the text (pages 22/23):

*Moreover, some participants expressed concerns about adopting the mSense system, mentioning potential anxiety from brain signal tracking and how it might be perceived in public. Two students expressed specific limitations regarding their reluctance to use the mSense system:*

*"I might use it once or twice, but I don't think I would wear the helmet regularly to collect this data." [Participant 28, Undergraduate Student, OEQ1]*

*"Knowing that the brain signals are being tracked when I am anxious would eventually fuel my anxiety." [Participant 45, Undergraduate Student, OEQ1]*

*In addition to these concerns, other participants highlighted further considerations:*

*"I believe I have a good understanding of my own mental state; yet, the headgear might provide additional insights." [Participant 78, Master’s Student, OEQ1]*

*"You might get funny looks on the train." [Participant 6, Researcher, OEQ1]*

*Overall, these perspectives highlight valuable feedback, touching on aspects such as practicality, social comfort, and emotional impact. While these insights point to areas for improvement, they also provide opportunities to refine the mSense system to enhance user-friendliness and accessibility for a broader audience.*
